# Blending insights and evidence: Co‐creating a community health worker intervention for postpartum patients with preeclampsia

**DOI:** 10.1002/pmf2.70084

**Published:** 2025-08-22

**Authors:** Jesse E. Rattan, K. T. Benson, T. H. Ashford, S. M. Miller, V. V. Shukla, C. P. Travers, A. A. Toluhi, M. B. Richardson, C. T. Longenecker, A. T. Tita, W. A. Carlo, R. G. Sinkey

**Affiliations:** ^1^ Department of Obstetrics & Gynecology University of Alabama at Birmingham Birmingham Alabama USA; ^2^ ConnectionHealth Birmingam Alabama USA; ^3^ PACT Washington District of Columbia USA; ^4^ University of Washington Seattle Washington USA

**Keywords:** adverse pregnancy outcomes, community health worker, human‐centered design, postpartum, preeclampsia

## Abstract

**Background:**

Preeclampsia is associated with an increased risk of future maternal cardiovascular disease and death. The American Heart Association (AHA) advocates for embedding place‐based interventions within communities to reduce long‐term cardiovascular health consequences. Yet a significant gap remains in proven strategies for postpartum risk reduction. Our objective was to design a pilot intervention that is now being tested in a randomized controlled acceptability and feasibility study.

**Methods:**

We leveraged our existing partnerships with a nonprofit community health worker (CHW) organization and our community advisory board to co‐create a postpartum CHW‐led intervention for people with preeclampsia. Using human‐centered design, we engaged in a workshop with people with lived experience of preeclampsia and other adverse cardiovascular pregnancy outcomes, CHWs, and clinicians. We employed client personas, an integrated service blueprint‐patient journey map, and other human‐centered design tools to develop a postpartum CHW‐led intervention.

**Results:**

We successfully adapted an existing perinatal CHW program to address the specific needs of postpartum individuals with preeclampsia. The resulting intervention pairs patients and their infants with CHWs who provide culturally tailored health education, emotional support, social service coordination, and community resource linkages during the first 12 weeks postpartum. Novel preeclampsia components include close blood pressure monitoring and facilitated connections to primary care providers focused on cardiovascular disease risk reduction.

**Conclusion:**

Human‐centered design tools supported a diverse group of patients and providers to rapidly design a pilot postpartum CHW intervention that supports patients with preeclampsia, which is being tested in our acceptability and feasibility trial (NCT06353256).

## 1 INTRODUCTION

Rates of maternal death among women in Alabama are among the highest in the United States and the developed world [1]. Preeclampsia is a significant contributor to overall maternal morbidity and mortality, and our tertiary care academic medical center in Alabama reports preeclampsia rates of 16%–20% among delivering patients, primarily due to maternal transfers requiring specialized neonatal care [2]. The long‐term implications of preeclampsia and other hypertensive disorders of pregnancy include a two‐ to fourfold elevated risk of future cardiovascular disease (CVD) and CVD‐related mortality [3]. Despite evidence of the long‐term harm of preeclampsia, and scientific guidance from the American Heart Association (AHA) and others advocating for improved postpartum strategies for people with preeclampsia and other adverse pregnancy outcomes (APOs), few clinical trials evaluate the impact of postpartum interventions that include evidence‐based cardiovascular risk‐reducing care [3].

To respond to this lack of evidence, our group launched the Alabama Womb2Heart Solution (AW2H) (NCT06353256), a randomized controlled acceptability and feasibility trial (*N* = 60) that pairs postpartum patients who have experienced preeclampsia or other APO with a community health worker (CHW) who supports them during the first 12 weeks after birth. If the pilot demonstrates acceptability and feasibility, a fully powered trial will test whether the intervention can improve short‐term and long‐term maternal cardiovascular health.

This distinct and separately funded pilot builds upon and leverages the infrastructure and expertise of AHA's Health Equity Research Network on Disparities in Maternal‐Infant Health Outcomes (P^3^ EQUATE). P^3^ EQUATE is a national, multi‐institutional initiative funded by the AHA to reduce inequities in maternal and infant outcomes, particularly among Black and Native American women and rural people. With an emphasis on community‐engaged, interdisciplinary research, this network of five projects is coordinated by the Center for Research in Women's Health at the University of Alabama at Birmingham and supports innovative strategies to improve access to quality care across the preconception, pregnancy, and postpartum continuum.

Within this network, the ongoing POPPY randomized controlled trial (RCT) (NCT06510075) is investigating whether a culturally tailored perinatal CHW model improves maternal and infant outcomes. POPPY CHWs provide emotional support, health education, mentoring, and connection to social services and community resources (e.g., housing, food, diapers, transportation, medication, school supplies) during pregnancy and the early postpartum period. We aimed to adapt the POPPY CHW model for our separate project, AW2H, to meet the distinct postpartum needs of individuals with preeclampsia or other APOs. Recognizing that interventions designed with end users are more effective, we united a group of clinicians, researchers, CHWs and people who had experienced an APO to co‐design the postpartum CHW intervention focused on short‐ and long‐term cardiovascular risk reduction for patients with preeclampsia and other APOs. See Annex S1 for a figure describing the relationship between P3 EQUATE, POPY, and AW2H.

To guide this co‐design process, we selected human‐centered design (HCD) as a practical and inclusive approach suited to the constraints and opportunities of our setting. HCD is a creative, participatory methodology increasingly used in healthcare to generate innovative, context‐sensitive solutions to complex challenges. HCD emphasizes designing interventions and systems with end users through iterative cycles of problem exploration, ideation, and rapid prototyping, grounded in empathy and co‐creation [4, 5]. The approach has been adopted to improve intervention design in clinical care, health education, and community‐based programs—particularly where time, funding, or stakeholder trust are limited [6, 7, 8]. One of the most widely recognized visual models of HCD is the Double Diamond framework, which outlines four phases: Discover, Define, Develop, and Deliver, reflecting the shift between divergent and convergent thinking used throughout the process [9]. In this study, we used a brief, community‐engaged HCD process to adapt an existing perinatal CHW intervention to address the postpartum needs of patients with preeclampsia.

## 2 MATERIALS AND METHODS

Given the practical constraints of limited time and funding, we selected HCD specifically for its flexibility and adaptability to rapid, resource‐constrained innovation. Unlike other formal, linear design processes, HCD allows for iterative problem‐solving even under tight timelines. Our use of a single, half‐day participatory workshop was intentional, since we aimed to build on an existing perinatal CHW intervention rather than design a program de novo. This compressed “diverge–converge” cycle enabled us to generate pragmatic solutions by leveraging preexisting clinical provider–community relationships and shared insight. Our approach builds on other HCD studies in clinical and public health contexts, where short design sprints have proven effective in co‐creating feasible, patient‐informed interventions under real‐world constraints [10, 11, 12]. Our diverse team conducted one “diverge–converge” cycle using the following human‐centered design tools paired with the most recent evidence about postpartum care of individuals with cardiovascular APOs, including preeclampsia:

- **Client personas**: We co‐developed two “client personas,” which are a method of representing and understanding user needs through the development of a composite user with a name and a vivid story [13]. To develop the client personas used in the design workshop, we used a collaborative synthesis process. Drawing on the lived experiences of our team's CHWs—several of whom are from the communities they serve—we created two composite personas that reflect archetypal clients they have supported. Together with our colleagues from ConnectionHealth, the community non‐profit supporting the CHW program, we synthesized these insights into fictional representative persona profiles with names, demographics, and rich contextual details. These profiles were refined collaboratively by the smaller pre‐workshop team (which included clinicians, researchers, and CHWs) and then reviewed and discussed during the workshop to center empathy and guide ideation. This process aligns with best practices in persona development [13] (see Annex S2).
- **An integrated service blueprint + patient journey map**: To expand and tailor the existing perinatal CHW service package, we used two well‐known human‐centered design tools—the service blueprint and the patient journey map. Service blueprinting is a simple participatory process that uses a matrix or diagram to display each step of service delivery and includes the people and process touchpoints that a client or patient interfaces with [14]. A journey map is a visual guide describing patient experiences as they interact with the healthcare system along their care journey [8]. We merged these two tools to create a matrix on the wall, using flipchart paper and markers to define current activities, explore patient experiences, and design new activities (see Annex S3 for our final blueprint‐journey map).
- **Impact‐Effort Matrix**: We prioritized ideas we generated using an Impact‐Effort 2 × 2 Matrix. An Impact‐Effort Matrix is a widely used tool in design and organizational planning that helps groups of people rapidly assess the relative value and feasibility of an idea. The matrix is structured as a 2 × 2 grid, with one axis rating the possible impact of an idea (i.e., the potential to improve an outcome) and the other axis rating the effort required to implement the idea (i.e., time, resources, complexity). The 2 × 2 grid creates four quadrants: (1) low effort–low impact, (2) high effort–low impact, (3) high effort–high impact, and (4) low effort–high impact, and allows the group to quickly analyze and rank generated ideas and prioritize the most strategic and meaningful ideas to take forward [15]. We again used sticky notes to generate ideas on the large matrix consisting of four squares on flipchart paper (see Annex S4 for the 2 × 2 Impact‐Effort Matrix we completed).

### 2.1 The co‐creation process

Our co‐design team included 11 individuals: three clinician‐researchers, one senior, experienced research coordinator, three CHWs/CHW leaders, one community health worker/fellow and one CHW‐researcher from ConnectionHealth, and two individuals with lived experience of an APO. The group was convened through existing academic–community partnerships to ensure diverse and relevant perspectives. Nine members identified as African American or Black—including one bilingual (Spanish‐English) member from Puerto Rico—and two identified as White. We structured the workshop to elevate community perspectives—centering lived experience in persona development, highlighting CHW‐led insights during journey mapping, and participatory facilitation that fostered inclusion. This stance informed not only our design choices, but also how we interpreted insights and prioritized elements of the final intervention prototype.

We intentionally scheduled the workshop to allow working parents to drop off children at school, commute to the workshop, and return for after‐school pickup. Breakfast and lunch were provided. Before the workshop, a smaller team iteratively developed two client personas that we used to anchor our design discussions. We opened with an overview of the workshop objectives, reviewed the tools, and conducted a group icebreaker. For our warm‐up, we briefly reviewed the current and emerging intervention evidence, generating key points together from the AHA scientific statements (see Table 1). Using flipcharts and markers, we then created a large service blueprint‐patient journey map template, spanning the eight CHW touchpoints from the POPPY project across the 12‐week postpartum period. For Activity One, we generated the current POPPY CHW activities across those postpartum touchpoints. For Activity Two, we broke into two groups and discussed the two patient personas we had jointly created before the workshop. Each group separately generated sticky notes with insights about what these individuals might be feeling and experiencing during the 12‐week postpartum period and placed these notes across the eight postpartum touchpoints. We then came together as one large group to highlight where there were synergies and divergences in insights across the two groups and discussed the gaps and opportunities to support the patient. During Activity Three, we reviewed and refined our evidence summary from the AHA scientific statement by clarifying and sharpening the key takeaways and then developed ideas about how to integrate the recommended interventions into the CHW package. During Activity Four, the team broke out into small groups again to generate potential CHW activities (on sticky notes) across each touchpoint, based on the results of Activity Two (insights) and Activity Three (evidence). As a large group, we discussed opportunities and challenges of integrating the preeclampsia/APO‐focused activities. We agreed that embedding preeclampsia and APO education in the social and emotional wrap‐around support offered by the CHWs fostered continuity and trust. Recognizing the persistent challenge of supporting postpartum moms navigating fatigue and overwhelm, we retained the POPPY model's patient‐driven visit schedule (in‐person, phone, video, or clinic‐based) to ease that burden and support flexibility and autonomy. An additional challenge we discussed was fragmented services—especially in primary care—which often prevent postpartum individuals from accessing support for their own health. Our team is actively working to address this within our health system. During our final activity (Activity Five), we collaborated as a large group to organize the potential CHW activities across a four‐quadrant Impact‐Effort Matrix to prioritize the most feasible and impactful activities. The logic of the Impact‐Effort Matrix helped us achieve rapid consensus on all of the key CHW activities and health promotion (which we considered low effort and high impact) drawn from our discussion of the current evidence. We ranked a critical part of both the intervention and usual care—provision of blood pressure cuffs and education on how to use them during hospital discharge—as high effort and high impact. We also quickly discussed two additional health promotion activities—incorporating postpartum “alert” bracelets and a take‐home educational refrigerator magnet into hospital discharge education but ranked them as high effort/resources and low impact and so did not prioritize them as part of the intervention. We then organized a smaller team to iterate further, refine, and plan logistical next steps (people, process, funding) on an online whiteboard and concluded our rapid design workshop (see Annex S5 for a detailed, step‐by‐step workshop agenda.)

**TABLE 1:** Evidence summary for CHWs.

The first year after birth is the **golden year**: a prime time to boost heart health for life and future pregnancies.
People with preeclampsia face **2–4 times higher risk** of high blood pressure and heart disease later in life
Blood pressure (BP) peaks 3–6 days after birth, then drops in the first 3 weeks—**close BP checks** are vital during this time.
**Help patients track BP** at home to catch problems early.
Teach new mothers the **warning signs of high blood pressure**: bad headaches, blurry vision, chest pain, breathing trouble, and swelling.
**Support breastfeeding**, which may lower a mother's future risk of high blood pressure.
Help find and set up a visit with a **primary care doctor** in the first year after birth.
Plan for **cholesterol testing** within the first year after birth.

## 3 RESULTS

The final intervention includes the original CHW program of emotional, social, and navigational support and novel activities for postpartum clients with preeclampsia or other APOs. CHWs closely guide clients, who have been discharged with a blood pressure cuff, to self‐monitor their blood pressure twice weekly from postpartum weeks one through three and once weekly during weeks four through 12, with further assistance with appointment scheduling with their maternity care provider if blood pressure is elevated. During postpartum week eight, CHWs help clients identify and schedule a visit to a primary care provider who will carefully track CVD risk factors and support CVD risk‐reducing strategies. In addition to educating on preeclampsia warning signs and the benefits of breastfeeding, CHWs also conduct targeted health promotion, during check‐ins (phone‐based or in‐person) beginning week three to week twelve, and discuss one to two elements of the AHA Life's Essential 8 Framework, with guidance on eating better, being more active, quitting tobacco/nicotine, getting healthy sleep, controlling cholesterol, and managing blood pressure [16]. After the check‐ins, CHWs send supportive Life's Essential 8 materials (e.g., tip sheets, infographics) via email or text to their clients.

We are now testing this intervention in our acceptability and feasibility pilot trial (NCT06353256). See Annex S3, our completed journey map‐service blueprint, that includes activities and educational talking points related to preeclampsia and APOs across the CHW touchpoints. See Annex S4 for a completed Impact‐Effort Matrix. See Annex S5 for a detailed, step‐by‐step workshop agenda, and Annex S6 for a visual representation of the program.

## 4 DISCUSSION

With modest resources and a compressed schedule, a mixed group of former patients, community members, CHWs, and clinical staff rapidly designed a comprehensive CHW package of support for postpartum people with preeclampsia. A future direction that emerged from our workshop is to design more detailed CHW support for infants who have experienced perinatal complications such as preterm birth or growth restriction related to maternal preeclampsia.

## 5 CONCLUSION

We successfully used a short cycle of human‐centered design to quickly co‐design our pilot intervention using both community insight and scientific evidence. These insights, along with data from our current pilot trial, will inform another cycle of the diverge–converge human‐centered design process, which will help us sharpen and refine the intervention as we prepare for our planned larger trial powered for key clinical outcomes.

## CONFLICT OF INTEREST STATEMENT

The authors declare no conflicts of interest.

## Supporting information



Supporting Information

Supporting Information

Supporting Information

Supporting Information

Supporting Information

Supporting Information

## Data Availability

The data that support the findings of this study are available on request from the corresponding author. The data are not publicly available due to privacy or ethical restrictions.
