# Supplementary material for: Blending insights and evidence: Co‐creating a community health worker intervention for postpartum patients with preeclampsia
Source: Pregnancy (Hoboken). 2025 Aug 22;1(5):e70084. doi: 10.1002/pmf2.70084 (PMC13344661; doi:10.1002/pmf2.70084)
Supplement: Supplementary file 1 [file PMF2-1-e70084-s001.pdf]

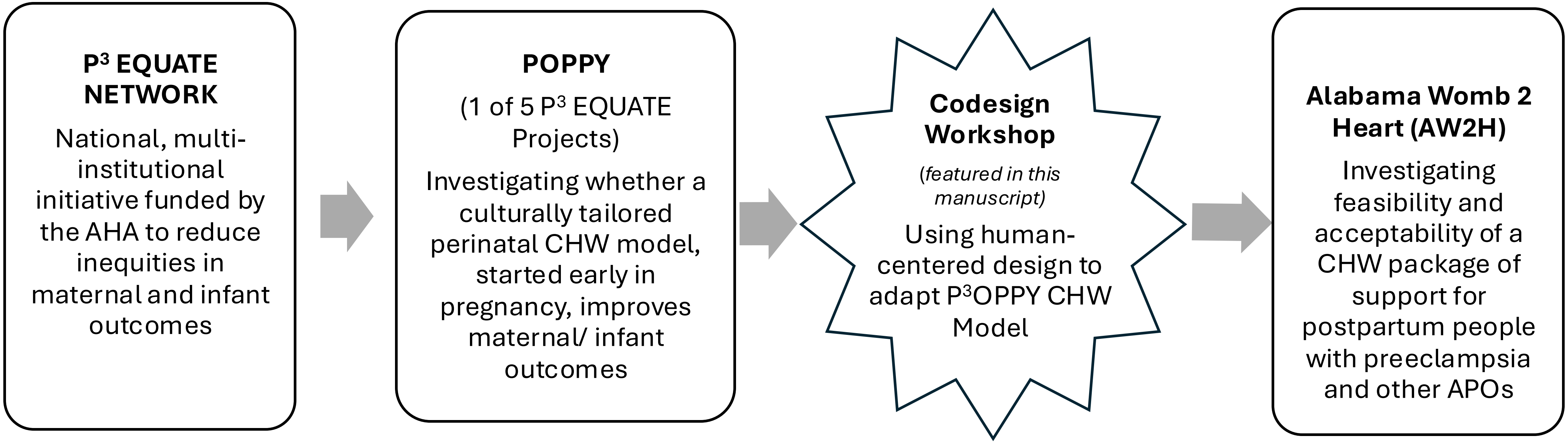

Annex 1: Figure describing the relationship between the P3 EQUATE Network, POPPY Project, codesign workshop featured in this manuscript, and AW2H CHW Project support for postpartum people with preeclampsia and other APOs

Abbreviation Key: AHA = American Heart Association; CHW = community health worker
