# Supplementary material for: Blending insights and evidence: Co‐creating a community health worker intervention for postpartum patients with preeclampsia
Source: Pregnancy (Hoboken). 2025 Aug 22;1(5):e70084. doi: 10.1002/pmf2.70084 (PMC13344661; doi:10.1002/pmf2.70084)
Supplement: Supplementary file 2 [file PMF2-1-e70084-s003.pdf]

Annex 3: Hybrid Journey Map and Service Blueprint Template

Journey Phase	Week 1/Early post-partum	Week 2	Week 3	Week 4	Week 5	Week 6-12
**CHW Touchpoint**	2-3	4	5	6	7	8-12
**Patient thoughts, feelings, needs**	Anxiety & exhaustion<br><br>Possible postpartum depression onset<br><br>Need to maintain normalcy for and mothering of other children<br><br>Possible breastfeeding challenges	May still feel overwhelmed<br><br>Possible postpartum depression<br><br>Could be feeling alone<br><br>Trying to get by day to day<br><br>Baby may be in the NICU/ mom may need to travel back and forth to NICU	Healthcare support might drop away<br><br>Possible postpartum depression<br><br>May experience financial strains including healthcare bills	Baby may still not sleep through night-continued fatigue especially with other kids or not enough support<br><br>Possible postpartum depression	Possible postpartum depression<br><br>Concerns about returning to work<br><br>Concerns about finding childcare<br><br>Still no time to focus on self	May have to return to work at 6 weeks PP<br><br>Once working, may have challenges with work schedule especially if baby is sick<br><br>If breastfeeding may worry about breastfeeding<br><br>May be interested in birth control
**Current CHW support activities**	Reminder to register baby for WIC & support with registering baby for health insurance;<br><br>Educate regarding Milk Banks and Diaper banks;<br><br>Assist with resources<br>Importance of breastfeeding for the baby's health<br><br>Ensuring the baby is feeding well and has regular wet and dirty diapers<br><br>Reminder of 2-week pediatric check-up	Help mom find free diapers and female hygiene products near them<br><br>Reminder to register baby for WIC & support with registering baby for health insurance<br><br>Educate regarding Milk Banks and Diaper banks.<br><br>Reminder of the first pediatric check-up<br><br>Infant safety education (safe sleep practices (back to sleep, firm	PP visit reminders<br><br>Assist with resources<br><br>Preventing child injuries education<br><br>Importance of immunizations and keeping up with the vaccination schedule	Dietary recommendations and physical activity<br><br>Stress management techniques & sleeping habits<br><br>Assist with resources<br><br>Importance of supervised tummy time for baby's development<br><br>Monitoring baby's growth and developmental milestones	Smoking education (incl. secondhand smoke and vaping)<br><br>PP visit reminder<br><br>Assist with resources<br><br>Immunization reminder<br><br>Effects of secondhand smoke on the baby<br><br>Support for smoking cessation<br><br>Resources for support in creating a healthy home	Home environment safety (conduct home visit if feasible)<br><br>Assist with resources<br><br>Continued importance of breastfeeding or formula feeding<br><br>Keeping baby's health records up-to-date<br><br>Maintain a heart-healthy diet rich in fruits, vegetables, and whole grains.<br><br>Incorporate moderate exercise, such as walking

		mattress, no soft bedding)<br><br>Contraception education if they are not currently using				Keeping up with pediatric check-ups and vaccinations<br><br>Engaging with baby through play and interaction for cognitive development<br>Overview of baby's health and development<br><br>Importance of ongoing pediatric care and immunizations<br><br>Exist study, assisting with resources for continued parenting and healthcare support
**Proposed activities based on #2 + #3 + AHA Scientific Statement on Postpartum Strategies for People with APOs**	Reminder of BP check 1- 2x/week for the first two weeks and share numbers with CHW (verbally)<br><br>Follow ACOG guidance for elevated BP: If hypertensive disorder of pregnancy, schedule a follow-up appointment within the week.<br><br>Discuss: Importance of continuing to monitor blood pressure<br><br>Discuss: Increased risk of CVD after APO & also opportunities to protect CVH<br><br>Be available to process APO and childbirth trauma	Reminder of BP check-up – at least 2x/week.<br><br>Follow ACOG guidance if BP is elevated.*<br>Empower patient to make follow up appointments as recommended by clinical provider.<br><br>For moms with gestational diabetes: encourage the 2-hour glucose test at PP visit	Continue home BP checks 1–2x/week. If BP has been consistently normal, transition to 1x/week.<br><br>Follow ACOG guidance if BP is elevated.*<br><br>Introduce elements of AHA Life's Essential 8: eating better; being more active; quitting smoking; health sleep; weight management; BP, cholesterol and blood sugar checks	Continue home BP Check-up - 1x/week if normal at Week 4.<br><br>Follow ACOG guidance if BP is elevated.*<br><br>Reinforce Importance of postpartum follow-up visits<br><br>Discuss 1-2 elements of AHA Life's Essential 8: eating better; being more active; quitting smoking; healthy sleep; weight management; BP, cholesterol and blood sugar checks	Continue home BP Check-up - 1x/week if BP is normal at Week 5.<br><br>Follow ACOG guidance if BP is elevated.*<br><br>Encourage continuing weekly BP monitoring. If BP was elevated at Week 4 or remains elevated, CHW should encourage the client to raise this with their provider during the **6-week postpartum visit** or sooner if symptoms develop.<br><br>Reinforce understanding of BP readings, symptoms of concern (e.g., severe headache, vision changes), and the importance of timely	BP Check-up - 1x/week if BP is normal during these weeks.<br><br>Follow ACOG guidance if BP is elevated.*<br><br>Encourage continuing weekly BP monitoring throughout this period. If BP is still elevated, CHW reinforces the importance of follow-up with a **primary care provider (PCP)** for ongoing management and cardiovascular risk assessment.<br><br>Discuss 1-2 elements of AHA Life's Essential 8: eating better; being more active; quitting smoking; healthy sleep; weight management;

	Support breastfeeding as a way to reduce the risk of long-term high blood pressure.				clinical follow-up.<br><br>CHW provides ongoing support and coaching to prepare for the postpartum check-up.<br><br>Discuss 1-2 elements of AHA Life's Essential 8: eating better; being more active; quitting smoking; healthy sleep; weight management; BP, cholesterol and blood sugar checks	BP, cholesterol and blood sugar checks<br><br>Assist in scheduling regular appointments with a primary care provider to monitor blood pressure and overall heart health: Encouraging them/empowering them to seek and schedule the PCP appointment within 6 months of delivery.<br><br>Support mom's confidence to discuss with PCP experience of APO, cholesterol, blood pressure and blood sugar checks<br><br>For PP visit: Counsel no estrogen-containing birth control if hypertensive or treated hypertension
* ACOG guidance[1] for management of elevated BP: If BP is ≧ 160/110, CHW counsels patient to be promptly seen by a provider; if BP is between 140/90-159/109, patients are encouraged to notify provider.						
Abbreviation Key: CVD = cardiovascular diseases, CVH = cardiovascular health, PP = postpartum, NICU = neonatal intensive care unit, WIC = Special Supplemental Nutrition Program for Women, Infants, and Children,						

Reference

1. Gestational Hypertension and Preeclampsia: ACOG Practice Bulletin, Number 222. *Obstet Gynecol*. Jun 2020;135(6):e237-e260. doi:10.1097/aog.0000000000003891
