# Supplementary material for: Blending insights and evidence: Co‐creating a community health worker intervention for postpartum patients with preeclampsia
Source: Pregnancy (Hoboken). 2025 Aug 22;1(5):e70084. doi: 10.1002/pmf2.70084 (PMC13344661; doi:10.1002/pmf2.70084)
Supplement: Supplementary file 3 [file PMF2-1-e70084-s004.pdf]

Annex 2: Client Personas for CHW Clients Experiencing Preeclampsia

1. Sarah is a 37-week pregnant patient with preeclampsia. She is excited about becoming a mother and a devoted partner to her husband, who works long hours as a construction worker to support their growing family. They already have a 3-year-old son eagerly awaiting his baby sister's arrival. However, Sarah feels overwhelmed by the prospect of managing two young children while her husband works. Sarah and her family live in a modest apartment in a neighborhood where resources can be scarce because they are in a lower income bracket. She has no car, so she relies on public transportation or occasional rides from friends and family. Unfortunately, their apartment has a mold problem, and when they notify the manager, they are reluctant to deal with it due to their low resources. They are faced with the difficult decision of whether to move or stay.

   Sarah had been working as a cashier at a local grocery store until her doctor advised her to take maternity leave due to preeclampsia. The loss of income has added another layer of stress to her already challenging situation. She is most worried about making ends meet. A few weeks later, Sarah is admitted to the hospital to give birth and deliver a healthy but preterm baby girl. The next day, she is approached by someone who asks if she would like to participate in a CHW support program and Sarah agrees. However, she is still unsure about CHW, and her husband seems apprehensive about having a stranger assist his wife.

   The CHW meets Sarah, her husband, and the baby in the NICU to introduce herself as a companion for her postpartum journey.

2. Meet London, a 32-year-old mother of four who was transferred from her provider in Greenville in her third trimester due to preeclampsia to UAB Medical Center to give birth. London's life is

full of responsibilities and challenges, further complicated by living in a healthcare desert. She does not own a car, which makes it difficult for her to attend medical appointments and access healthcare facilities. Being a single mother and looking for a job, London is solely responsible for managing the household and caring for her children. Since the children's father is only sometimes around, she must also fulfill his role. London's journey with her premature baby has been an emotional rollercoaster. The abrupt preterm labor, the long and stressful transfer to UAB, and her baby’s subsequent NICU stay have left her physically exhausted and mentally drained. She is juggling the demands of caring for her premature son, worrying about his health, and trying to maintain some semblance of normalcy for her other children. A CHW program coordinator approached London to participate in the CHW program. London sees this as an opportunity to receive support and agreed to participate. She will go back home to Greenville but is hopeful that a CHW can support her there.
