# Supplementary material for: Blending insights and evidence: Co‐creating a community health worker intervention for postpartum patients with preeclampsia
Source: Pregnancy (Hoboken). 2025 Aug 22;1(5):e70084. doi: 10.1002/pmf2.70084 (PMC13344661; doi:10.1002/pmf2.70084)
Supplement: Supplementary file 4 [file PMF2-1-e70084-s005.pdf]

Annex 5: Detailed Workshop Agenda for Rapid Design of Community Health Worker Intervention

Approach & Duration	Activity
Preparation:<br>1 week and 1 day before	● A smaller team works on developing patient personas and shares with the larger group for feedback. Ideally, this team includes patients/users and community health workers.<br>● Send out agenda, project overview, and workshop objectives ~1 week prior to the workshop. Review design principles on a 30-minute call or, if time constraints, share principles asynchronously<br>● Identify a large enough room to ensure team members can comfortably sit and stand in a circle and break out into small groups.<br>● Ensure adequate amounts of flipchart paper, large sticky notes, and markers in different colors.<br>● Prepare a visual journey map + service blueprint: draw a large grid across multiple pieces of flipchart paper per the template.
Participatory welcome, introductions, objectives and norms<br><br>30 minutes	● Welcome, quick introduction icebreaker<br>● Describe workshop objectives and ask for any last-minute feedback<br>● Rapidly discuss and develop group norms for the day<br>● Describe the workshop process and give an overview of activities and flow
Participatory warm-up and content refresher<br><br>15 minutes	● Refresh content knowledge on preeclampsia. Use a game like Bingo or Jeopardy to brush up on knowledge.
Activity 1: Describing the current services (large group)<br><br>30 minutes	● "Walk through" the service blueprint on the wall as a large group.<br>● Individuals write out activity bullets on sticky notes that are currently in the CHW intervention package. As each person finishes them, put them up in the correct cells/boxes across the service blueprint touchpoints.<br>● In the large group discuss and organize the current CHW activities that are now on sticky notes on the service blueprint.
Activity 2: Building empathy through discussions of client personas and generate patient experiences (small and large group work)<br><br>40 minutes	● In the large group, read and discuss patient personas briefly together as a large group<br>● Break up into 2 groups.<br>● Discuss each persona. Imagine each individual's experience and journey across the 12 postpartum weeks. How are they feeling? What are they doing? What are the joys? What are the challenges?<br>● Work individually for about 10-15 minutes, share reflections in their small groups, then each group posts their sticky notes on the service blueprint-journey map.<br>● Conduct a "gallery walk" as a large group (people walk in front of the flipcharts) to review the patient journey sticky notes on the service blueprints. Everyone reviews silently for 5-10 minutes.

	● Discuss patient journey per the sticky notes. No need to talk through everything. Highlight where group agrees, does not agree or where there are gaps.<br>● Note big gaps or clear opportunities to address challenges or build on opportunities.
Break<br>15 minutes	● Take a quick brain and body break. Offer coffee, tea, and snacks.
Activity 3:<br>Content refresher on evidence-based preeclampsia interventions<br><br>20 minutes	● Briefly walk through a summary of evidence-based interventions for people with postpartum preeclampsia.<br>● Discuss how these recommendations might be integrated into the CHW package.
Activity 4:<br>Generate potential activities in small groups<br><br>30 minutes	● Split back up into small groups to identify possible activities on sticky notes. These can be wild/generative. Don't hold back ideas. There is time to prioritize in the next activity. Include how evidence-based interventions can be included. *Alternative: if there are time constraints, you can do this activity as a large group*<br>● Put potential solutions on sticky notes and add them to the service blueprint.<br>● Briefly do a gallery walk as a large group, discussing and clarifying the activities generated by the two groups. Note synergies as well as gaps. Cluster ideas that are similar and synthesize. Add ideas that may emerge during the discussion on sticky notes.
Activity 5: Converge on top-rated activities using 2X2 impact and effort matrix<br><br>30 minutes	● Optional activity: "Dot voting" using stickers or markers, participants vote for their top 5 activities.<br>● As a group, discuss each activity and place on an Impact-Effort Prioritization Matrix<br>● Focus on the subset of activities that fall in Cell 1, then Cells 2 & 3<br><br>Cell 1: Higher Impact, Lower Effort | Cell 2: Higher Impact, Higher effort<br>Cell 3: Lower Impact, Lower Effort | Cell 4: Lower Impact, Higher Effort

Impact-Effort Prioritization Matrix (from Activity 5):

Cell 1:<br>Higher Impact<br>Lower Effort	Cell 2:<br>Higher Impact<br>Higher effort
Cell 3:<br>Lower Impact<br>Lower Effort	Cell 4:<br>Lower Impact<br>Higher Effort

Close<br><br>15 minutes	• Summarize top activities<br>• Discuss next steps (can use an online whiteboard)<br>• Lunch together
