# Supplementary material for: Blending insights and evidence: Co‐creating a community health worker intervention for postpartum patients with preeclampsia
Source: Pregnancy (Hoboken). 2025 Aug 22;1(5):e70084. doi: 10.1002/pmf2.70084 (PMC13344661; doi:10.1002/pmf2.70084)

Annex 4: Impact-Effort Matrix

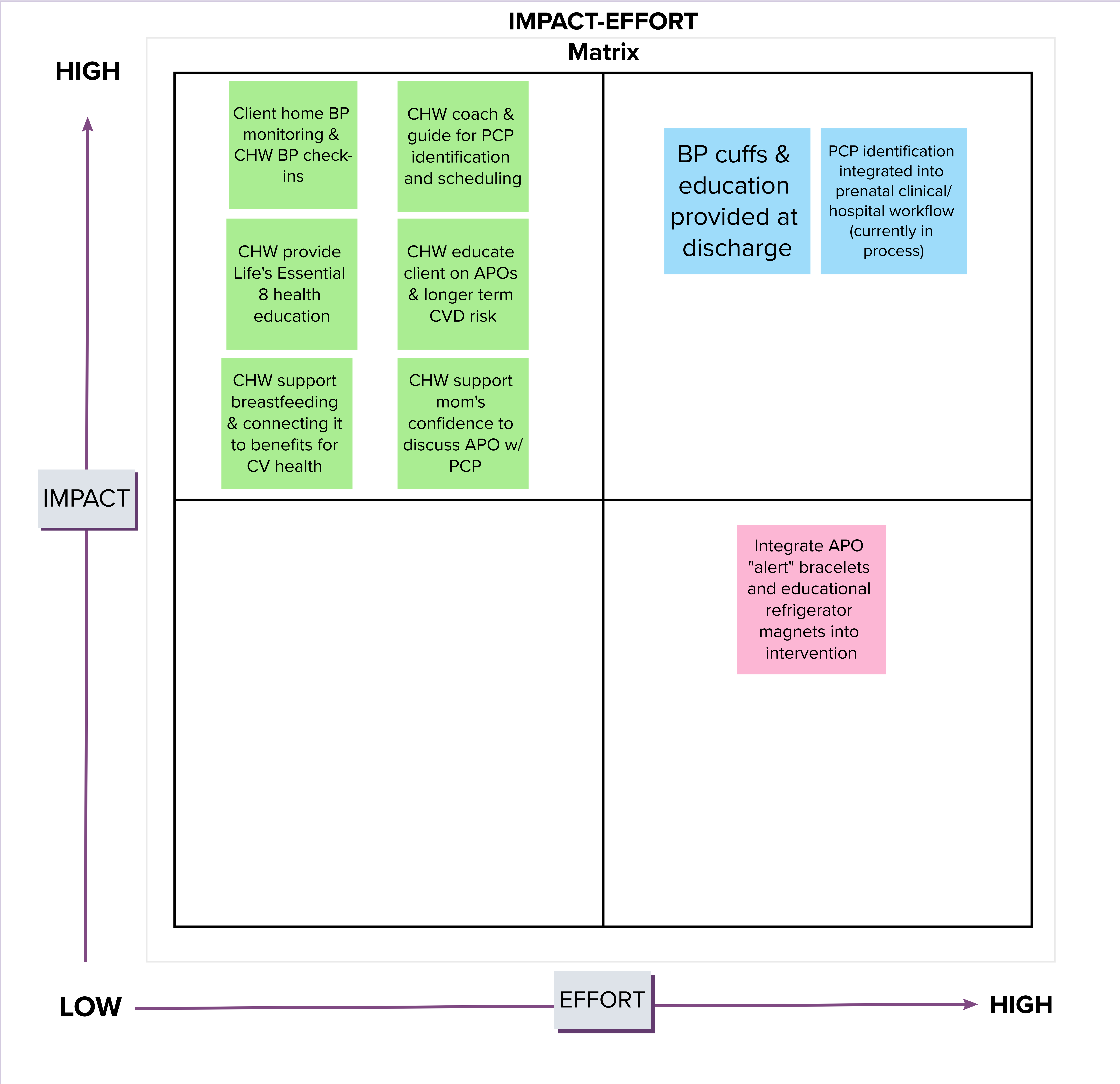

Supplement: Supplementary file 5 [file PMF2-1-e70084-s006.pdf]
